# Supplementary material for: Correlation between the Skin Permeation Profile of the Synthetic Sesquiterpene Compounds, Beta-Caryophyllene and Caryophyllene Oxide, and the Antiedematogenic Activity by Topical Application of Nanoemulgels
Source: Biomolecules. 2022 Aug 10;12(8):1102. doi: 10.3390/biom12081102 (PMC9405972; doi:10.3390/biom12081102)
Supplement: Supplementary file 1 [file biomolecules-12-01102-s001.zip › biomolecules-1823534-supplementary.pdf]

SUPPLEMENTARY MATERIAL

Correlation between the skin permeation profile of the synthetic sesquiterpene compounds, beta-caryophyllene and caryophyllene oxide, and the antiedematogenic activity by topical application of nanoemulgels

Patrícia Weimer [1], Tainá Kreutz [1], Renata P. Limberger [1], Rochele C. Rossi [2]; Ádley A. N. de Lima [3], Valdir F. Veiga Jr. [4], Bibiana V. de Araújo [1], and Letícia S. Koester [1,*]

[1] Programa de Pós-Graduação em Ciências Farmacêuticas, Faculdade de Farmácia, Universidade Federal do Rio Grande do Sul (UFRGS), Porto Alegre 90610-000, Rio Grande do Sul, Brazil

[2] Programa de Pós-Graduação em Nutrição e Alimentos, Universidade do Vale do Rio dos Sinos (UNISINOS), São Leopoldo 93022-000, Rio Grande do Sul, Brazil

[3] Programa de Pós-Graduação em Ciências Farmacêuticas, Departamento de Farmácia, Universidade Federal do Rio Grande do Norte, Natal 59012-570, Brazil

[4] Instituto Militar de Engenharia (IME). Rio de Janeiro 22290-270, Rio de Janeiro, Brazil.

[*] **Correspondence**: leticia.koester@ufrgs.br; Tel.: +55-51-33085278; Fax: +55-51-33085437

Supplementary Equations

Determination of retention index in chemical characterization of C. multijuga oleoresin

Eq. (S1)

$$RI (x) = 100 Pz + 100 [(RT (x) - RT (Pz)) / (RT (Pz+1) - RT (Pz))]$$

Where: RI (retention index); Pz (number of alkane carbons before the peak of interest); Pz+1 (retention time of the alkane after the peak of interest); RT (x) (peak sample retention time to be identified); RT (Pz) (retention time of the alkane before the peak of interest).

Determination of irritation score in Hen's egg chorioallantoic membrane test (HET-CAM)

Eq. (S2)

$$IS = [5 \times (301 – H\ sec)/300] + [7 \times (301 – V\ sec)/300] + [9 \times (301 – C\ sec)/300]$$

Where: IS (irritation score); H sec (start second of hemorrhage effect); V sec (start second of vasoconstriction effect); C sec (start second of coagulation effect). Classification: IS 0 - 0.9: non irritant; IS 1.0 – 4.9: slight irritant; IS 5.0 – 8.9 moderate irritant; IS 9.0 – 21.0 severe irritant.

Determination of edema inhibition in arachidonic acid-induced mouse ear edema model

Eq. (S3)

$$\text{Edema inhibition (\%)} = \left[1 - \left(\frac{REt - LEt}{REc - LEc}\right)\right] x\ 100$$

Where: RE (weight of right ear, in grams); LE (weight of left ear, in grams); t (treatment/vehicle groups); c (negative control group – acetone).

Supplementary Tables

Table S1 – Concentrations of β-caryophyllene (CAR) and caryophyllene oxide (OX) applied to the *in vivo* assay

**Treatment group**	**mg CAR/ear**	**mg OX/ear**
BN	-	-
CAR	3.60	-
NCAR-1	0.90	-
NCAR-2	1.80	-
NCAR-3	3.60	-
OX	-	2.00
NOX-1	-	0.50
NOX-2	-	1.00
NOX-3	-	2.00
COP	3.60	2.00
NCOP-1	0.90	0.50
NCOP-2	1.80	1.00
NCOP-3	3.60	2.00

BN – blank nanoemulgel; NCAR – nanoemulgel of β-caryophyllene; NOX – nanoemulgel of caryophyllene oxide; NCOP – nanoemulgel of *C. multijuga* oleoresin.

Table S2 – Chemical composition of *Copaifera multijuga* oleoresin (COP)

Peak	Compound	MW (g/mol)	RT (min)	RI calc.	RI lit.	%Area
1	δ-elemene	204.351	22.009	1331	1335	0.29
2	α-copaene	204.351	23.656	1368	1374	7.22
3	β-elemene	204.351	24.395	1385	1389	3.46
4	β-caryophyllene	204.351	25.567	1411	1417	36.51
5	γ-elemene	204.351	26.195	1426	1434	0.58
6	α-humulene	204.351	26.936	1444	1452	4.52
7	γ-muurolene	204.351	27.976	1468	1478	5.20
8	Germacrene D	204.351	28.122	1472	1484	1.15
9	α-muurolene	204.351	29.005	1493	1500	1.13
10	γ-cadinene	204.351	29.528	1506	1513	2.04
11	δ-cadinene	204.351	29.916	1516	1522	1.87
12	Germacrene B	204.351	31.203	1548	1559	1.65
13	Caryophyllene oxide	220.351	32.336	1577	1582	21.57
14	Humulene epoxide II	220.351	33.341	1603	1608	1.77
15	Junenol	222.37	33.688	1612	1618	2.56
16	Tau-cadinol	222.37	34.733	1640	1640	2.65
17	α-muurolol	222.37	34.880	1644	1644	1.88
18	α-cadinol	222.37	35.219	1653	1652	3.49
19	NI	-	57.902	-	-	0.47
				**Total identified**		**100.01**
				Monoterpene hydrocarbons		-
				Oxygenated monoterpens		-
				Sesquiterpenes hydrocarbons		65.620
				Oxygenated sesquiterpenes		31.27
				Other compounds		3.12

MW: molecular weight; RT: retention time; RI: retention index; Calc.: calculated; Lit.: literature; NI – not identified.

Supplementary Figures

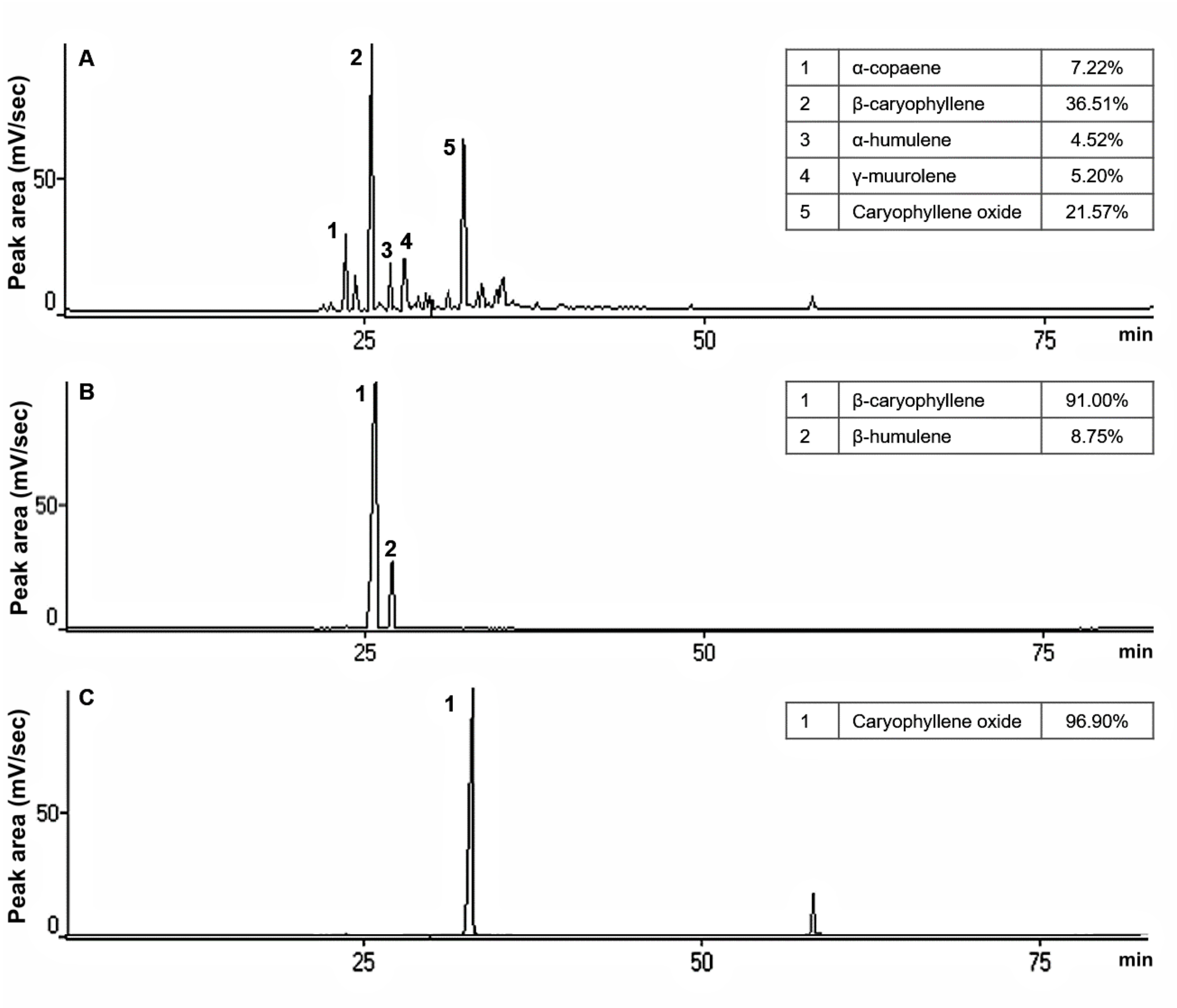

Figure S1 – GC-MS Total ion chromatograms of: (A) sesquiterpenes from *C. multijuga* oleoresin (COP), (B) β-caryophyllene (CAR); and (C) caryophyllene oxide (OX)

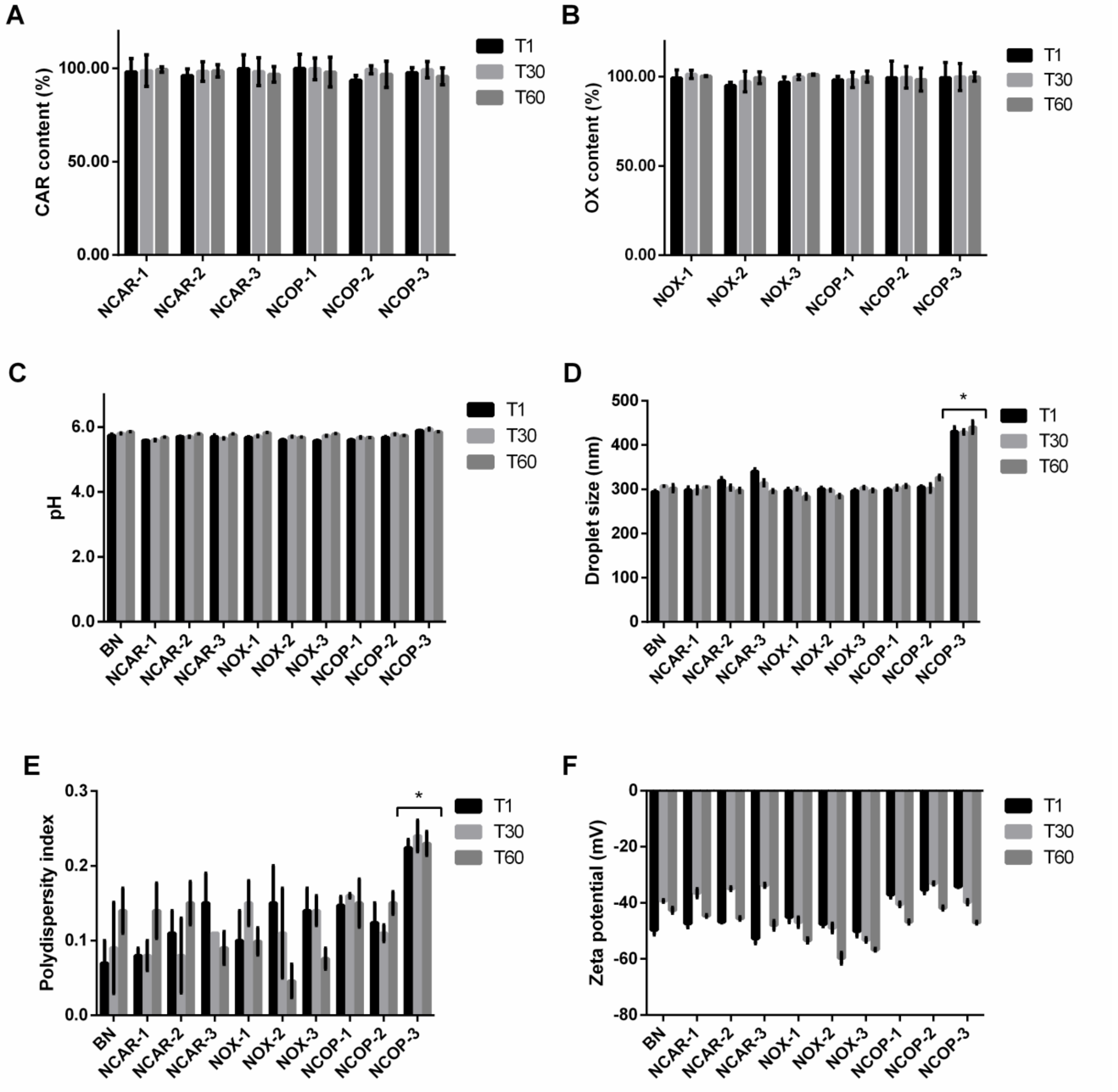

Figure S2. Characterization of nanoemulgels and storage stability (n=3). Nanoemulgels of β-caryophyllene (NCARs), caryophyllene oxide (NOXs), and *C. multijuga* oleoresin (NCOPs) as well as a blank nanoemulgel (BN) were assessed at 1 (T1), 30 (T30), and 60 (T60) days. β-caryophyllene content **(A)**; Caryophyllene oxide content **(B)**; pH **(C)**; Droplet size **(D)**; Polydispersity index **(E)**; Zeta potential **(F)**. *Statistically different from other groups ($p<0.05$). The results are expressed as mean ± SD of three independent experiments.

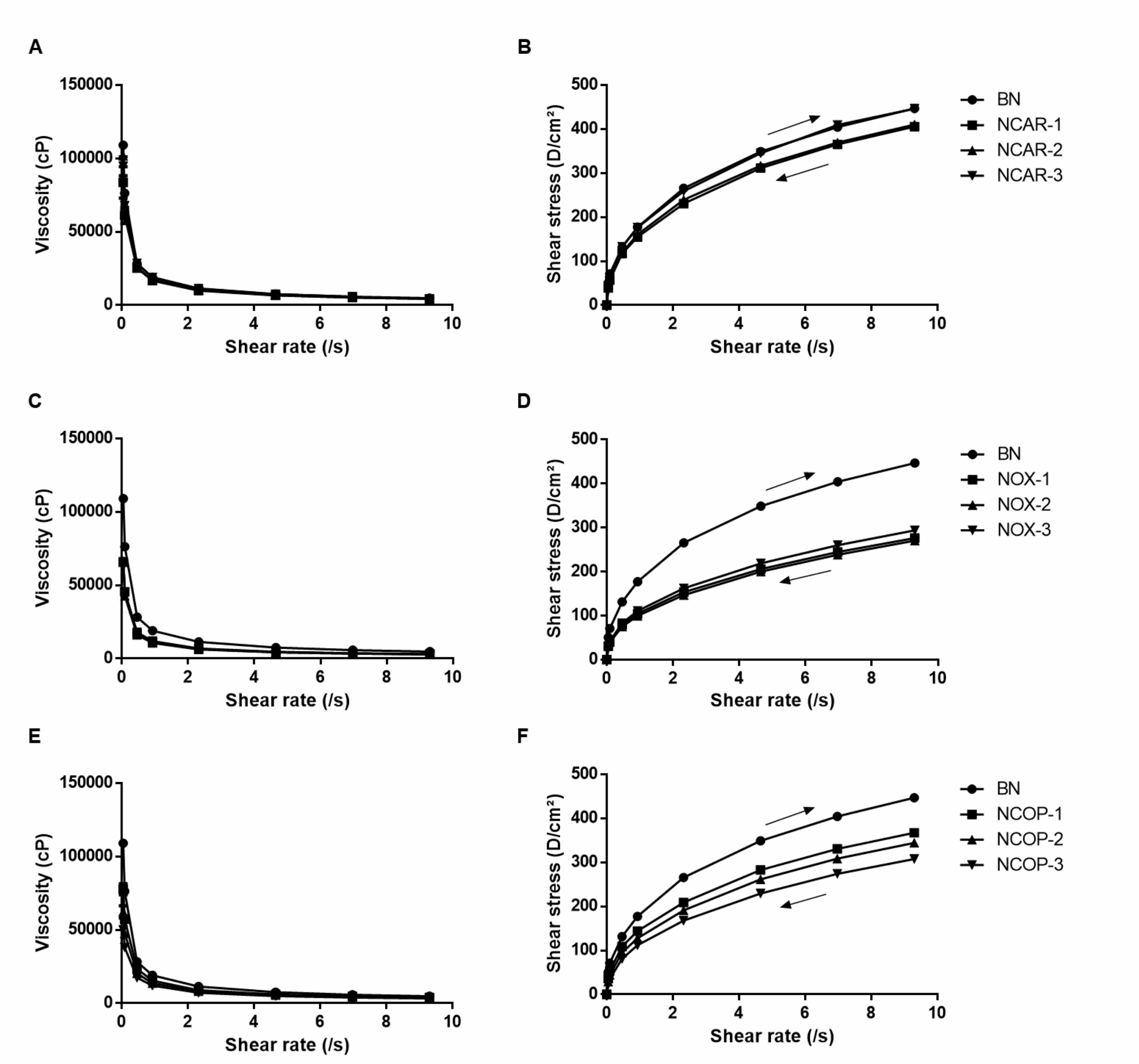

Figure S3 – Rheograms of developed nanoemulgels compared to blank nanoemulgel (BN). Nanoemulgels of β-caryophyllene (NCARs) **(A, B)**; nanoemulgels of caryophyllene oxide (NOXs) **(C, D)**; nanoemulgels of *C. multijuga* oleoresin (NCOPs) **(E, F)**. Nanoemulgels were sheared at repeated increasing and decreasing rates of shear, in triplicate, and arrows indicate the upcurves (→) and downcurves (←). The results are expressed as mean ± SD of three independent experiments.

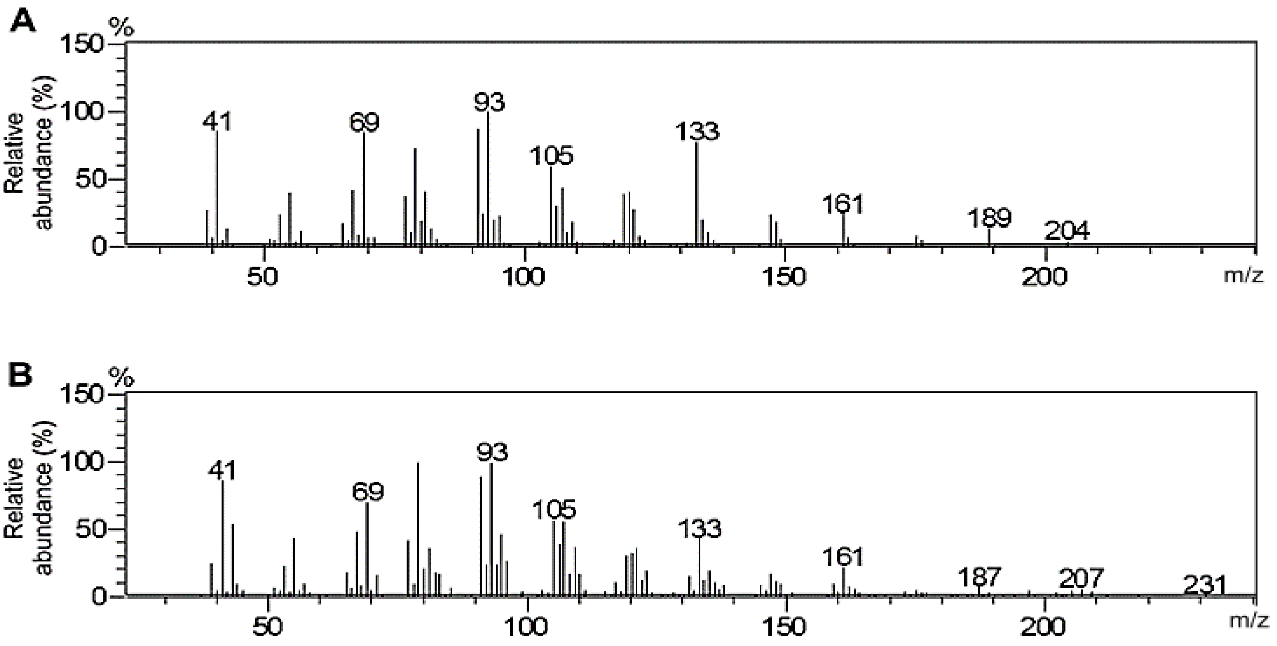

Figure S4 – Representative mass spectrum of β-caryophyllene **(A)** and caryophyllene oxide **(B)** after extraction from skin sample of the permeation assay.

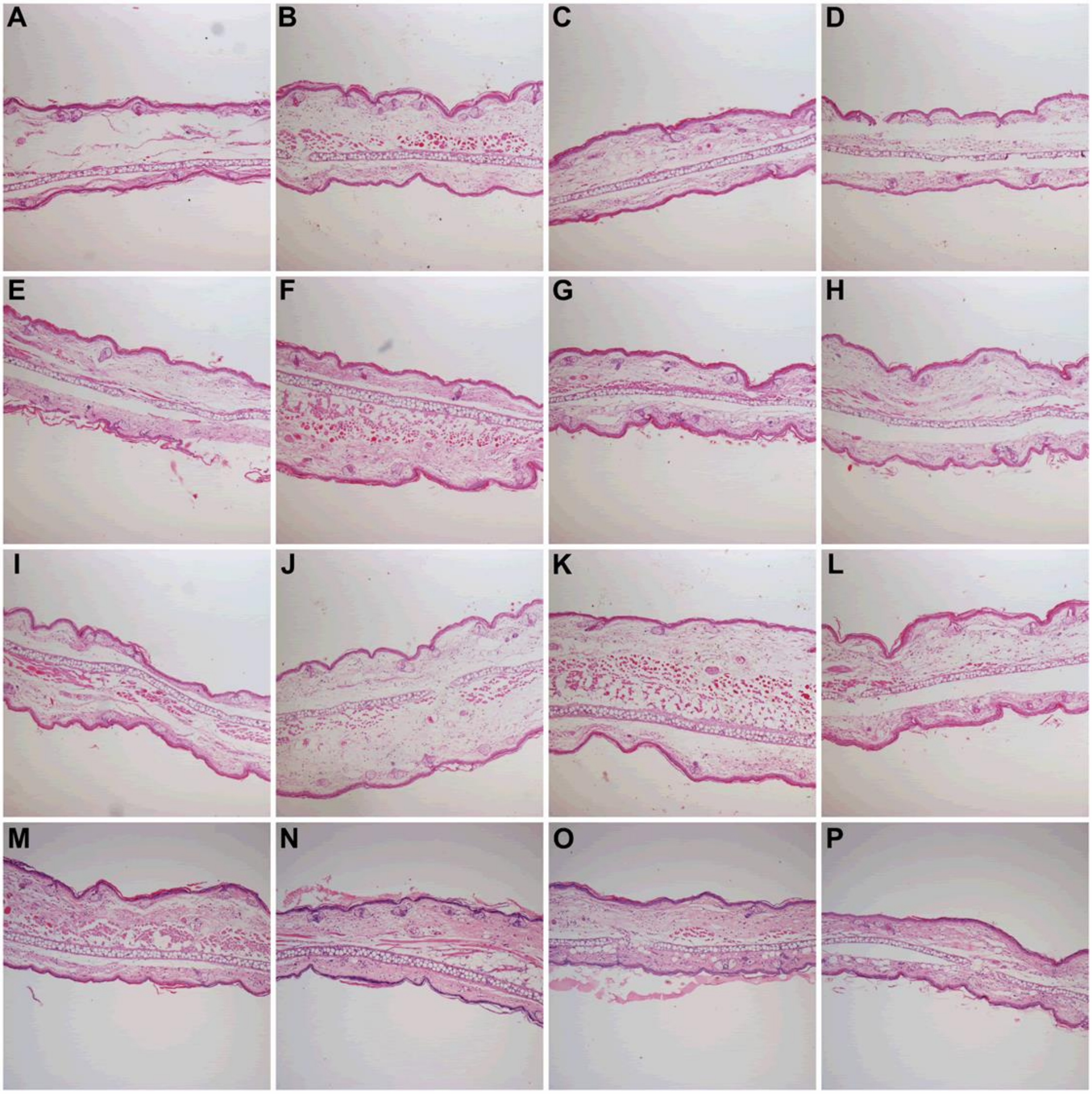

Figure S5 – Hematoxylin-eosin (H&E) staining of mouse ears sections after arachidonic acid-induced edema. Photomicrographs examined under optical microscope with 100x magnification. Groups: **(A)** Left ear (without induced edema); **(B)** Negative control – acetone; **(C)** Positive control - Indomethacin; (**D)** Blank nanoemulgel - BN; **(E)** β-caryophyllene - CAR; **(F)** NCAR-1; **(G)** NCAR-2; **(H)** NCAR-3; **(I)** *C*. multijuga oleoresin - COP; **(J)** NCOP-1; **(K)** NCOP-2; **(L)** NCOP-3; **(M)** Caryophyllene oxide - OX; **(N)** NOX-1; **(O)** NOX-2; **(P)** NOX-3.

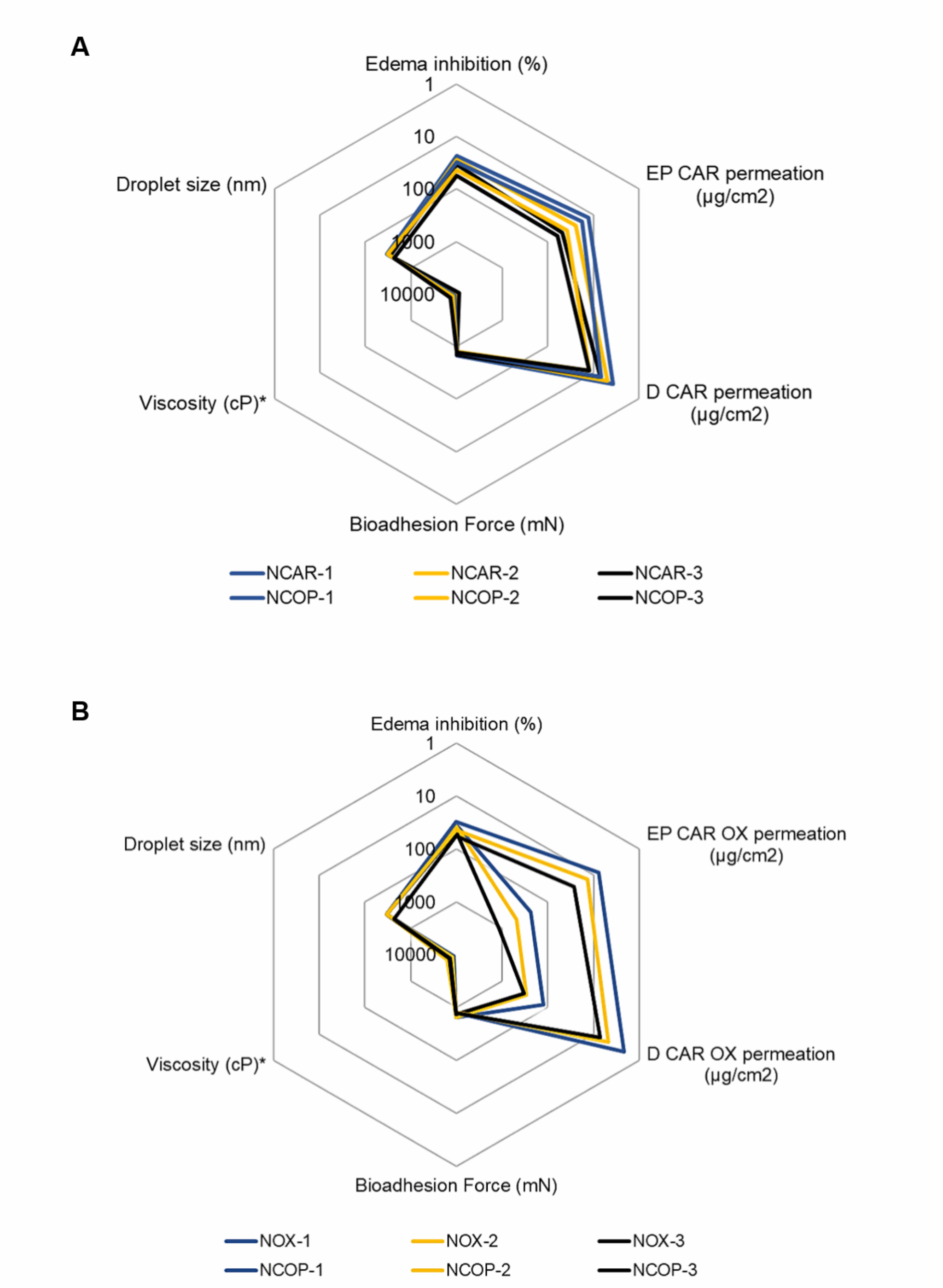

Figure S6 – Radar chart of nanoemulgel characteristics (droplet size, viscosity *at shear rate of 2.325/s, and bioadhesion force, variables on logarithmic scale), edema inhibition, and total amount of bioactive substances permeated in the epidermis and dermis. Comparison between NCARs and NCOPs **(A)**. Comparison between NCARs and NCOPs **(B)**. Same colors in each graph represent equivalent concentrations of CAR and OX in nanoemulgels (blue - lower concentrations, yellow - medium concentrations, and black - higher concentrations). Abbreviations: CAR - β-caryophyllene; OX – caryophyllene oxide; COP – *C. multijuga* oleoresin; N – nanoemulgel; EP – epidermis, D- dermis.
